# Knowledge Regarding Reproductive Tract Infection Among Ever Married Females of Reproductive Age Group in Rural Tamil Nadu

**DOI:** 10.7759/cureus.52885

**Published:** 2024-01-24

**Authors:** Surya Balakrishnan, Balabaskaran S, Saravana moorthy M, Kavin Kannan

**Affiliations:** 1 Community Medicine, Panimalar Medical College Hospital and Research Institute, Chennai, IND; 2 General Medicine, Government Vellore Medical College Hospital, Vellore, IND

**Keywords:** rural areas, ever-married females, public health education, knowledge level, reproductive tract infection

## Abstract

Background

Reproductive tract infection (RTI) is an overgrowth of the normal flora of the reproductive tract. It is an iatrogenic infection caused by unhygienic practices like unsafe abortion. Lack of knowledge plays a major role among the factors associated. The District Level Health Survey (DLHS)-4 reported that the knowledge about RTI among the rural population of Tamil Nadu is 8 percent. It is thus necessary to know about their knowledge regarding RTI.

Aim

To assess the knowledge regarding reproductive tract infection among the ever-married rural women in the reproductive age group in Kancheepuram district, Tamil Nadu.

Settings and design

A cross-sectional study was conducted in the rural Kancheepuram district of Tamil Nadu.

Materials and methods

The sample size calculated was 330. Using multi-stage random sampling, a population proportion to the calculated sample size was used. A standardized questionnaire was used for data collection. Ethical approval was obtained. Statistical analysis used SPSS -21.0 was used for statistical purposes. A chi-square test was applied for significance. P-value <0.05 is considered significant.

Results

Among 330 females, 166 (50.3%) presented with any symptom of RTIs in the past three months, and 300 (90.9%) have heard about RTIs, with the main source of information being health education by the health care workers (155, 46.9%); 9.1% (31) females had no or poor knowledge regarding the RTIs.

Conclusions

Because of a lack of knowledge, RTI was prevalent among women in the rural community who were of reproductive age. Regular health education should be given to women who are fertile in order to increase their understanding.

## Introduction

According to the estimation of the World Health Organization (WHO), approximately 340 million fresh instances of treatable sexually transmitted infections (STIs) emerge each year. This count excludes human immunodeficiency virus (HIV) and other viral STIs like hepatitis B, genital herpes, and genital warts, which are not curable. Among the curable STIs, the most prevalent ones are gonorrhoea, syphilis, chlamydia, and trichomoniasis. These infections collectively impose a significant health burden and elevate the chances of HIV transmission [1].

Reproductive tract infection (RTI) is the infection of the reproductive tract, majorly involving sexually transmitted infections, overgrowth of the normal flora of the reproductive tract, and iatrogenic infection due to improper procedures, unhygienic delivery practices, and unsafe abortion. Every year, thousands of women die due to or as a sequel to a reproductive tract infection [2].

Among the factors that are associated with RTIs, knowledge and lack of awareness about the disease play a major role, while unhygienic practices, inadequate health facilities, poverty, religious obstacles, and hormonal factors are also involved [3], which in turn could be a reason for less knowledge and awareness related to health issues. It is thus necessary to know about their knowledge regarding RTI [4]. In India, the annual incidence of RTIs and sexually transmitted diseases is projected at 5%, or approximately 40 million, every year [5]. According to the National Family Health Survey-4 report, 89.5% of the rural women of the reproductive age group follow good menstrual hygiene, especially the age group of 15-24 years, of which only 15.6% knew about RTI and its spread [6].

The DLHS-4 conducted during 2012-2013 reported that knowledge about RTIs among the rural population of Tamil Nadu is 8%. The questionnaires conducted in the national language and english in households and with ever-married women (ages 15-49) were framed. The ever-married women’s questionnaire, which is part of the questionnaire, contains information on women’s characteristics, maternal care, immunization and childcare, contraception and fertility preferences, and reproductive health, including knowledge about HIV/AIDS. The questionnaires enclosing details about the knowledge of RTI were considered for the study [7].

Developmental activities spread and influence the general population, which may be a reason for low awareness related to health issues. It is thus essential to know about the knowledge and practices regarding RTI [8].

This study aims to comprehensively evaluate the level of knowledge and understanding about RTIs among ever-married women within the reproductive age group in the rural setting of Tamil Nadu. Through an in-depth exploration of knowledge, attitudes, and practices concerning reproductive health, this research strived to elucidate the prevailing awareness levels, potential gaps in comprehension, and the socio-demographic factors influencing these insights. By focusing on rural regions within Tamil Nadu, the study endeavored to offer valuable insights into the specific needs and challenges faced by this demographic, with the aim to contribute to the formulation of targeted interventions and educational initiatives to enhance reproductive health outcomes.

## Materials and methods

Methodology

The present study was conducted within the rural field practice area of Chettinad Hospital and Research Institute, affiliated with Chettinad University. This community-based quantitative cross-sectional study focused on investigating reproductive health awareness among females in the area. The target demographic encompassed the female population within the area, totaling 19,065 individuals. From this population, a specific subset comprising 5,062 females falling within the reproductive age group was identified for potential inclusion in the study. Sample selection for the study was methodically executed, drawing from this subset to ensure a representative sample reflective of the broader population. The determination of the sample size was calculated based on a comprehensive analysis of previous literature [9-11]. Leveraging an average knowledge prevalence of approximately 24% regarding RTIs among ever-married women from these previous studies, a rigorous statistical calculation was done using the formula: n = 4pq/l2​, where ‘n’ represents the sample size, ‘p’ signifies the prevalence extracted from previous studies, ‘q’ is derived as (100 - p), and ‘l’ denotes the allowable error margin. The computed estimated sample size derived from this calculation amounted to 292 participants. Incorporating a provision for a 15% potential non-response rate due to factors such as non-cooperation or unavailability, the sample size was prudently adjusted and rounded to 330 participants. The sampling methodology adopted a multi-stage random sampling technique, ensuring a proportional representation of the population characteristics within the selected sample size. Exclusion criteria were meticulously applied, excluding females under 18 years and above 49 years of age, antenatal and postnatal mothers, as well as post-menopausal women. Upon securing informed written consent from the participants, a structured face-to-face interview was conducted. To ensure the questionnaire's reliability and relevance, a pilot study was executed with 30 participants before the commencement of the primary study. The pre-structured and pre-tested questionnaire comprehensively covered key variables such as age, educational attainment (ranging from primary school to graduate level and including the illiterate category), religion, socio-economic status, family type, marital status, knowledge assessment, and specific RTIs experienced within the preceding three months (Appendix Figure 1-5).

Ethical consideration

The ethical underpinning of this study was solidified through formal approval obtained from the Chettinad Institutional Ethical Committee at Chettinad University (IHEC number: 23/IHEC/3-16). Prior to participation, informed consent was diligently acquired from all individual respondents, ensuring their understanding of the study's objectives, procedures, and the voluntary nature of their involvement. Participant information sheets were discreetly maintained, upholding strict confidentiality to safeguard the privacy and anonymity of each participant. Adherence to ethical principles, including respect for persons, beneficence, justice, and non-maleficence, was paramount throughout every phase of the study's execution. In alignment with the risk assessment strategy outlined in the Indian Council of Medical Research (ICMR) guidelines, the present study was categorized as falling within the minimal risk category. Stringent measures were implemented to mitigate any potential risks or discomfort to participants.

Data entry and analysis

Data collection was meticulously carried out utilizing Epi Info software for entry, ensuring accuracy and consistency. The subsequent analysis was conducted employing SPSS version 21.0 software. Various statistical parameters such as frequency, mean, and standard deviation were calculated to comprehensively assess the dataset. The application of the chi-square test allowed for the evaluation of association among categorical variables within the data. A P-value of <0.05 was used to denote statistical significance. 

## Results

Table 1 projects the status of knowledge on RTI among the participants

**Table 1:** Status of knowledge on reproductive tract Infection among participants

Knowledge regarding reproductive tract infection	Status of knowledge	Number of females (N)	Percentage (%)
	Good	300	90.9
	Poor	30	9.1

Among the participants, 90.9% had adequate knowledge concerning RTI, whereas 10.1% had poor knowledge concerning RTI.

Out of the 330 participants, 166 (50.3%) reported experiencing at least one symptom of reproductive tract infection within the past three months. Health education delivered by healthcare workers emerged as the primary source of information about RTIs, as seen in 155 individuals (46.9%).

Regarding knowledge pertaining to RTIs, it was observed that vulval itching stood out as the most commonly recognized symptom, acknowledged by 37.3% of participants. Additionally, 57.6% of respondents identified the texture and color of the discharge as curdy white, signifying a prevalent awareness of this specific characteristic.

Table 2 shows the association of socio-demographic factors with knowledge of RTI.

**Table 2:** Association of socio demographic factors with the knowledge of reproductive tract infection * represents a statistically significant p-value (less than 0.05).

Variables	Categories	Knowledge about RTI		Chi -Square	p Value
		Good	Poor		
Age	18-27	72 (24)	3(10)	7.121	0.028*
	28-37	144(48)	12(40)		
	38-49	84(28)	15(50)		
Education	Primary school	51(17)	5(16.7)	10.674	0.049*
	Middle school	30(10)	2(6.7)		
	High school	69(23)	10(33.3)		
	Higher secondary	51(17)	3(10)		
	Graduate	57(19)	1(3.3)		
	Illiterate	42(14)	9(30)		
Religion	Hindu	211(70.3)	22(73.3)	0.231	0.891
	Christian	61(20.3)	5(16.7)		
	Muslim	28(9.4)	3(10)		
Socio-economic status	Upper	9(3)	0(0)	12.216	0.016*
	Upper Middle	45(15)	4(13.3)		
	Lower middle	125(41.7)	9(30)		
	Upper lower	86(28.7)	17(56.7)		
	Lower	35(11.6)	0(0)		
Type of family	Nuclear	180(60)	14(46.7)	2.105	0.349
	Joint	72(24)	9(30)		
	Three generation	48(16)	7(23.3)		
Marital status	Married	272(90.7)	27(90)	0.897	0.639
	Widowed	17(5.7)	1(3.3)		
	Divorce	11(3.6)	2(6.7)		

Among the 330 study participants knowledgeable about RTIs, the highest awareness was observed among individuals aged 28-37 (48%), while the lowest was noted in the 18-27 age group (24%). A significant association was evident in the analyses, reflected by a p-value of 0.014. In terms of education, participants who completed high school exhibited higher RTI awareness (23%), whereas those with a higher secondary education demonstrated comparatively lower knowledge (10%). This disparity yielded a notable association with a p-value of 0.049. RTI knowledge was notably prominent among Hindus (70.3%), although statistical analysis did not reveal a significant association.

Exploring the link between knowledge about RTIs and socioeconomic status, the highest awareness was found among the lower middle class (41.7%), contrasting sharply with the upper class, where only 3% demonstrated familiarity. This discrepancy was statistically significant, with a p-value of 0.016.

The participants living with a spouse showcased a higher knowledge percentage (90.7%) compared to unmarried individuals, although no significant association was established. The association between RTI knowledge and type of family was found to be insignificant, with the majority residing in nuclear families (60%) and the fewest in three-generation families (16%).

## Discussion

RTIs among ever-married women hold significant importance for various reasons. It allows for the prompt recognition of symptoms, facilitates timely medical intervention, and prevents the exacerbation of infections. Comprehensive knowledge empowers women to adopt preventive measures, including practicing proper hygiene and safe sexual practices, thereby minimizing the risk of infection spread and associated complications. This awareness plays a pivotal role in making informed decisions related to family planning, pregnancy health, and fertility concerns, ensuring well-considered choices and the pursuit of appropriate healthcare. Furthermore, it contributes to enhancing overall life quality by alleviating discomfort, enabling women to share accurate information, fostering community awareness, and reducing stigma surrounding reproductive health topics. Ultimately, this knowledge equips women to take charge of their health and positively influence the well-being of their families and communities.

In the context of understanding reproductive tract infections (RTIs), the findings from this study revealed a significant level of awareness among the study population, with 90.9% demonstrating familiarity with the symptoms associated with RTIs. This comprehensive grasp of RTI symptoms within this cohort underlines the importance of knowledge dissemination and awareness campaigns in promoting reproductive health. Comparatively, an analogous study conducted in the Surendranagar district [12] reported a baseline understanding of the disease at 64%. The stark difference between these percentages accentuates the variability in RTI awareness across different demographic settings and emphasizes the critical role of regional, targeted educational initiatives to enhance knowledge and understanding about RTIs. In a study assessing the knowledge, care-seeking behavior, and prevalence of RTIs among tribal women in Himachal Pradesh, India, findings indicated an infection prevalence of 22.7%. The primary complaint reported was lower abdominal pain at 37.10%, followed by vaginal discharge at 31.60%. Notably, only 40% of the affected women sought treatment for these infections [11]. In a study conducted by Rizwan et al., the prevalence of these infections was determined to be 43.60%. Remarkably, the study highlighted that 11% of these females had never even heard of RTIs, indicating a notable gap in awareness within this demographic [13]. In another study conducted by Ratnaprabha et al., the prevalence of significant symptoms of reproductive tract infection was 29.15%. Regarding awareness about the infection, it was too low among the rural population, which suggested the need for health programs to promote the prevention of the disease [10]. Pham et al. conducted a study to estimate the prevalence of STIs and RTIs among women who were married and in the age group between 18 and 49 years and to explore the knowledge of healthcare providers in rural Vietnam. In this study, 78% of the participants were not aware of symptoms of RTI, and the knowledge score assessed was only 6.5 out of 40 [14]. Among the participants in the study, 90.9% had an awareness of RTIs. The primary source of this knowledge was health education programs facilitated by healthcare workers, accounting for 52% of participants, while 45% gained their understanding from consulting doctors for their specific health concerns. Additionally, a minority, 3% of participants, acquired information through media outlets, notably newspapers. Notably, the age group of 28-37 years exhibited the highest knowledge percentage at 48%, followed by the 18-27 and 38-39 age groups. The level of education directly influenced RTI awareness, with higher educational attainment correlating with increased knowledge. Similarly, the participants' socio-economic status was directly linked to their level of knowledge about RTIs. Our study highlighted strong associations between knowledge levels and the participants' age groups, educational qualifications, and socio-economic standings. However, no significant associations were found between knowledge about RTIs and factors such as religion, family type, or marital status among the participants.

Strengths and limitations

This was a community-based study. The study findings were based on a face-to-face interview using a pre-tested questionnaire. However, it was conducted with a limited sample size, and a lack of in-depth exploration of the reasons for poor knowledge is needed to create intervention-based strategies at the community level. A qualitative study was further planned based on the findings of the present study.

## Conclusions

In conclusion, the prevalence of RTIs among females of reproductive age in rural communities underscores a concerning trend. This prevalence primarily arises from a concerning pattern where symptomatic individuals refrain from seeking treatment for their complaints. This avoidance is often rooted in several underlying factors, notably a lack of awareness regarding the symptoms of the disease, the pervasive stigma surrounding it, and the prevalence of inadequate menstrual and personal hygiene practices. To address these multifaceted challenges, a proactive approach is imperative. This necessitates the implementation of consistent and targeted health education initiatives specifically tailored for females in the reproductive age bracket in rural areas. These initiatives should emphasize not only the identification of RTI symptoms but also encourage and motivate individuals to seek proper medical management for any concerning complaints. The focus should be more on early diagnosis and treatment of RTIs, an adequate supply of RTI test kits, and drugs required to treat RTIs. Moreover, a comprehensive focus on educating both reproductive-age women and adolescents about menstrual hygiene and personal care practices is crucial to curbing the prevalence of RTIs. Health education emerges as a pivotal tool, bridging the knowledge gap, particularly among individuals from lower socio-economic backgrounds and early adulthood. By empowering individuals with adequate knowledge, especially regarding symptom recognition and appropriate management, these educational interventions present an immediately feasible approach to alleviating the burden of RTIs within the community. 

## Figures and Tables

**Figure 1:**
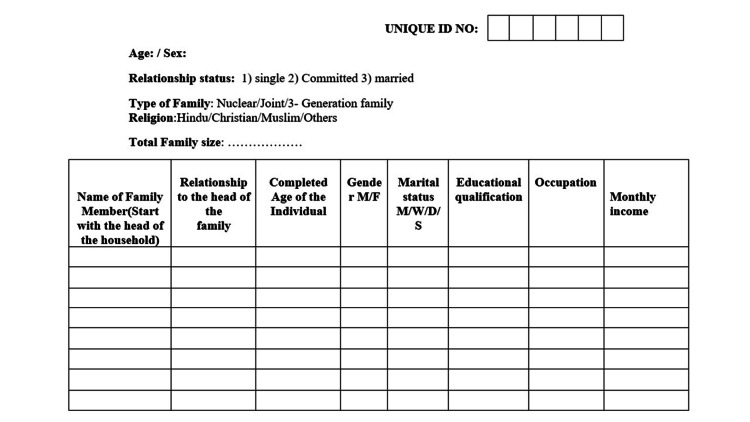
Questionnaire part 1

**Figure 2:**
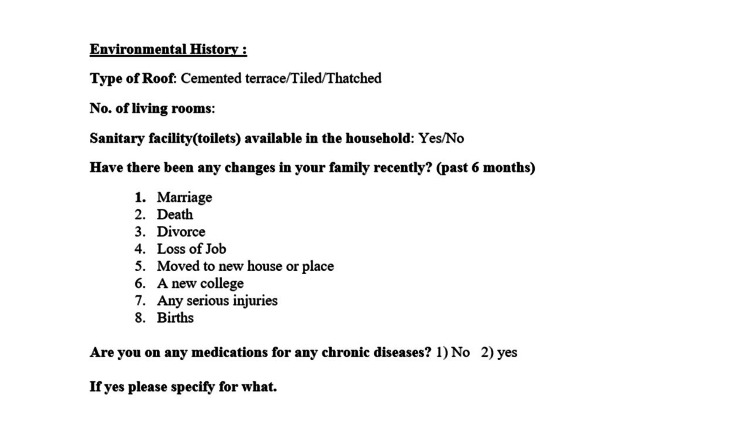
Questionnaire part 2

**Figure 3:**
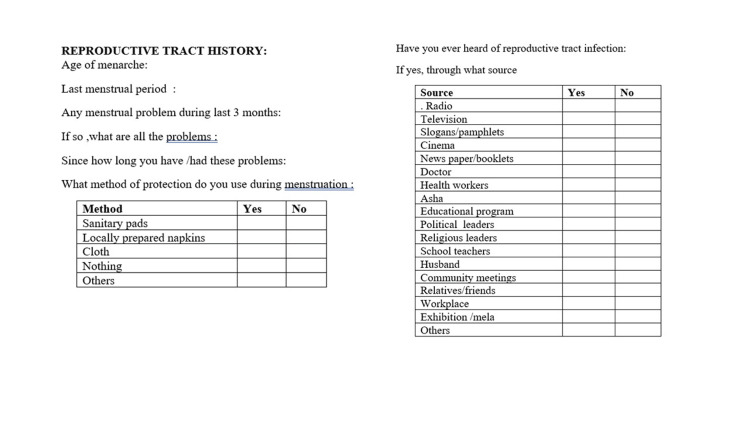
Questionnaire part 3

**Figure 4:**
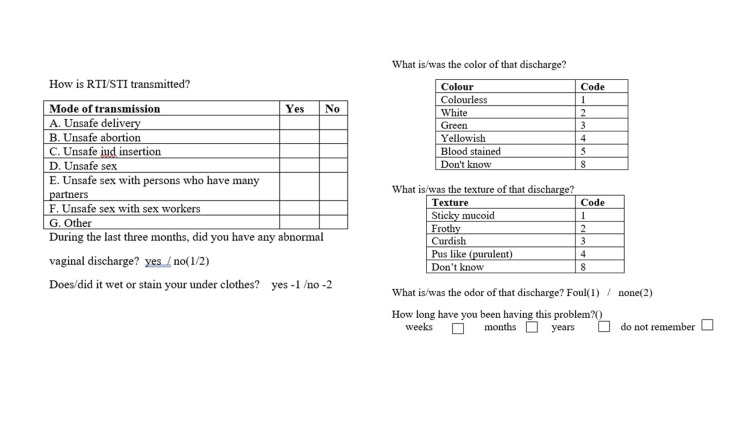
Questionnaire part 4

**Figure 5:**
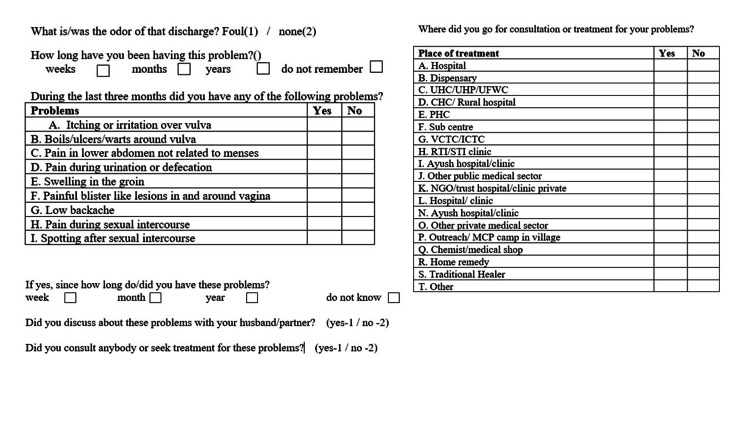
Questionnaire part 5 UHC: Urban health centre, UHP: Urban health post, UFWC: Urban family welfare centre, CHC: Community health centre. PHC: Primary Health Centre, VCTC: Voluntary counseling and testing center, ICTC: Integrated counseling and testing center, RTI: Respiratory tract infection, STI: Sexually transmitted infection, MCP: Mother and Child Protection.

## Appendices

Figure 1-5 shows the questionnaire used in the study
